# Knowledge, attitudes and perceptions of antibiotic use and resistance among patients in South Africa: A cross-sectional study

**DOI:** 10.4102/sajid.v34i1.118

**Published:** 2019-08-28

**Authors:** Elise Farley, Dena van den Bergh, Renier Coetzee, Annemie Stewart, Tom Boyles

**Affiliations:** 1 Public Health and Family Medicine Department, Faculty of Health Sciences, University of Cape Town, Cape Town, South Africa; 2 Division of Infectious Diseases and HIV Medicine, Department of Medicine, Groote Schuur Hospital University of Cape Town, Cape Town, South Africa; 3 School of Pharmacy, Faculty of Natural Sciences, University of the Western Cape, Cape Town, South Africa; 4 UCT Clinical Research Centre, Faculty of Health Sciences, University of Cape Town, Cape Town, South Africa; 5 Department of Medicine, University of the Witwatersrand, Johannesburg, South Africa

**Keywords:** Antimicrobial resistance, Antibiotic resistance, South Africa, Knowledge, Attitudes, Perceptions, Patient

## Abstract

**Background:**

Antibiotic resistance (ABR) is a global health crisis. We conducted a cross-sectional survey to describe South African patients’ (*n* = 782) ABR knowledge, attitudes and perceptions (KAP), differences in KAP between public (*n* = 379, 48%) and private (*n* = 403, 52%) practice respondents and associations between attitudes, perceptions and knowledge scores.

**Methods:**

Knowledge scores (15 questions) were placed into low (0% – 53%) and high (> 54%) categories (below and above overall mean). Comparisons were conducted using chi-squared and *t*-tests.

**Results:**

Of all respondents, 72% believed it was the human body that becomes resistant to antibiotics, 66% stated that antibiotics are good for treating viruses and 25% of patients believed that people should be given antibiotics on demand. Mean knowledge scores were lower in public sector respondents (public 45%, s.d. 15%; private 60%, s.d. 30%; *p* ≤ 0.001). Public practice patients with high knowledge scores were more likely to report both negative KAP (antibiotic prescriptions justify doctors’ visits, scientists will discover new antibiotics) and protective KAP (finishing a course of antibiotics, antibiotics do not treat all illnesses). Private practice patients with high knowledge scores were marginally less likely to report negative KAP (wanting antibiotics after long illnesses or when very sick) and more likely to report protective KAP (antibiotics have side effects and are a strong treatment).

**Conclusion:**

Our study shows differences in KAP by practice type and that greater knowledge increases the likelihood of protective attitudes, perceptions and behaviours.

## Introduction

Antibiotic resistance (ABR) is an acute global public health threat.^1^ It is estimated that 700 000 people die from ABR infections in a year.^2^ The mechanisms of ABR are well described and include enzymatic degradation of antibacterial drugs, alteration of bacterial proteins that are antibacterial targets and changes in bacterial membrane permeability to antibacterial agents.^3^ The development of ABR is accelerated by the over prescription and misuse of antibiotics.^1,4,5,6,7,8,9,10,11^

Antibiotic stewardship is critical in the efforts to slow down ABR, and patients play a fundamental role in stewardship.^12^ One of the core elements of antibiotic stewardship programmes in health care is education of prescribers, dispensers and patients.^13^ For the latter group, their misuse of antibiotics is in part because of a lack of understanding of the true causes of ABR. A recent systematic review on the public’s understanding of ABR showed that 88% believe ABR originates within the human body, rather than within the microbial organism itself.^14^ Patients also lack an understanding of their role in controlling ABR with studies showing that 87% of patients blamed ABR on general practitioners for over prescribing antibiotics^15^ and that limiting ABR was out of their control.^16^ A lack of understanding of the ramifications of antibiotics overuse leads to overprescribing, as prescribers often feel pressurised to prescribe antibiotics because of patient expectations.^5^ Seventy-four per cent of respondents who asked for an antibiotic were prescribed one in a United Kingdom (UK)-based study.^17^ A recent South African study showed that 66% of antibiotic prescribers in primary care feel pressurised to prescribe antibiotics.^18^

The knowledge of the burden of ABR in the African continent is inadequate because of sparse surveillance data^19^ and limited monitoring of prescribing behaviours.^2,6,14,20,21,22,23,24,25,26,27^ The South African department of health published an implementation plan for the antimicrobial resistance strategy framework in 2015 which states that surveillance data in South Africa shows resistance in all major types of bacteria that cause infection.^28^ The main strength of the South African system are the network of laboratories which provide surveillance data, a strong (if small) group of experts, and infection control systems and practices.^28^ The main weaknesses are a lack of understanding of the true burden of resistance, incomplete and variably reported data on antimicrobial consumption, limited training opportunities on ABR, lack of accountability and limited research on ABR in South Africa.^28^ A key strategic enabler in the plan is to raise awareness of ABR in the community. This is to be implemented by developing a communication strategy and methods to target the community, then implementing a campaign for the public on ABR including the improvement of patient knowledge, attitudes and perceptions (KAP) through activities such as awareness weeks.^28^ Currently, there are limited data from South Africa to guide this process.

In order to contribute to the tailoring of ABR awareness programmes in South Africa, we conducted a cross-sectional study among patients of public and private sector primary health care structures to assess the KAP of patients on antibiotic use and resistance.

## Methods

We conducted a cross-sectional survey of patients attending public and private primary health care facilities in South Africa. From April 2016 to May 2017, we collected data from 26 private practice primary care medical and dental centres and six public sector practices. A convenience sampling approach was used; adult (≥ 18 years old) patients attending private sector practices were asked by reception staff to complete the paper-based survey when they arrived for consultations. Adult patients attending public sector facilities were approached by research staff and asked to complete the questionnaire. Paper-based answers were captured onto *SurveyMonkey* (SurveyMonkey Inc., San Mateo, California, USA) by the research staff.

The structure and content of the survey were based on those used in previously conducted studies^7^ and were adapted for our setting by discussion with infectious diseases specialists and general practitioners (GPs). The self-administered questionnaire (Appendix 1) recorded basic demographic information, knowledge, perceptions and beliefs about antibiotic use and resistance. Knowledge on ABR was tested through 15 questions that could be answered ‘yes’, ‘no’ or ‘unsure’. These questions were statements, all of which had a correct answer based on the most current biomedical knowledge. We calculated a ‘knowledge score’ for each respondent based on the number of correct answers given. Knowledge scores were calculated from the total number of questions answered by individual respondents rather than the total number of questions in the survey. We categorised all respondents based on the mean score of 53%, as similar studies have utilised this method of categorisation.^29,30,31,32^ The low (mean and below) knowledge categories were those who scored 0% – 53%, and the high (above mean) knowledge scores were those who scored 54% – 100%. Answers in the ‘unsure’ category of all behaviours or beliefs were grouped together with the ‘disagree’ category answers to get a binary outcome.

Mean knowledge scores and demographic characteristics were compared using *t*-tests. Chi-squared tests were conducted to test for differences in responses between private and public practice patients. We also stratified patient groups into above and below mean knowledge groups.

All analysis was conducted using Stata 14 (StataCorp. 2015. *Stata Statistical Software: Release 14*. College Station, TX: StataCorp LP).

## Ethical consideration

Ethical approval for this study was granted by the University of Cape Town, Human Ethics Research Committee (HREC REF: 722/2016 and 610/2015). Written informed consent was obtained from all respondents. Ethical clearance was issued by the University of Cape Town, Faculty of Health Sciences on 14 November 2016.

## Results

### Demographic characteristics

Seven hundred and eighty-two patients completed the survey: 379 (48%) in public practices and 403 (52%) in private sector facilities. Most respondents were female (*n* = 545, 73%). The largest age group was 25–34 years old (*n* = 190, 25%) and 44% (*n* = 326) of patients had completed secondary school. The gender division was similar between public and private practice patients (females: public *n* = 282, 74%; private *n* = 263, 72%; *p* = 0.47), and private sector patients were generally younger (*p* ≤ 0.001) and had a higher level of education (*p* ≤ 0.001) (Table 1).

**TABLE 1:** Demographic characteristics.

Variable	Total(*N* = 782)		Public (*N* = 379)		Private (*N* = 403)		Chi-square test
	*n*	%	*n*	%	*n*	%	
**Gender**							
Female	545	73	282	74	263	72	0.47
Male	199	27	97	26	102	28	
Missing	38	-	0	-	38	-	
**Age**							
< 25	83	11	89	24	101	27	< 0.001
25–34	190	25	68	18	92	24	
35–44	160	21	78	21	72	19	
45–54	150	20	61	16	55	15	
55–64	116	15	51	13	32	8	
> 65	58	8	31	8	27	7	
Missing	25	-	1	-	24	-	
**Highest level of education**							
Grade 1–8	90	12	85	23	5	1	< 0.001
Grade 9–11	173	23	148	39	25	7	
Matric	326	44	128	34	198	54	
University degree	106	14	2	1	45	12	
Postgraduate degree	47	6	13	3	93	25	
Missing	40	-	3	-	37	-	

### Beliefs and behaviours

Sixty-one per cent (*n* = 473) of respondents were concerned about ABR and realised that antibiotics will work less well in the future if they are overused now and 58.0% (*n* = 456) believed ABR will be costly to the world. Several adverse aspects of antibiotics were noted including 63.0% (*n* = 491) of respondents stating that antibiotics have a negative effect on the body’s natural balance, 82.0% (*n* = 637) stating that they are a strong form of medication and should only be taken when absolutely necessary and 53.0% (*n* = 415) specified they get worried when antibiotics are prescribed, as they prefer not to take them.

Differences can be seen in the beliefs and attitudes between public and private sector patients. Public sector patients were more likely to believe that people should be given antibiotics on demand (public *n* = 146, 39.0%; private *n* = 47, 12.0%; *p* ≤ 0.001). Public sector patients were also more likely to believe that scientists will discover new antibiotics when the old ones stop working (public *n* = 261, 69.0%; private *n* = 164, 43.0%; *p* ≤ 0.001).

There were differences between the groups in terms of behaviours in that private sector patients displayed less harmful behaviours for ABR development. Private sector patients were less likely to report having taken antibiotics meant for a friend or family member (public *n* = 85, 23%; private *n* = 37, 10%; *p* ≤ 0.001), saving unused antibiotics to use at a later time (public *n* = 97, 26%; private *n* = 64, 18%; *p* ≤ 0.001), giving their own antibiotics to a friend or family member (public *n* = 84, 22%; private *n* = 46, 13%; *p* ≤ 0.001) and exaggerating symptoms in order to get antibiotics (public *n* = 72, 19%; private *n* = 30, 8%; *p* ≤ 0.001).

### Knowledge scores

Knowledge scores were normally distributed; overall, the mean knowledge score was 53% (s.d. 19.0%). All of the questions were correctly answered by 2 (0.3%) respondents, and 8 (1.0%) respondents answered all of the questions incorrectly. The mean number of non-responsive participants per knowledge question was 13 patients (2.0%). Public sector patients had a lower mean knowledge score compared to private practice patients (45%, s.d 15% vs. 60%, s.d 30%; *p* ≤ 0.001). Notably, only 13% of all respondents (all *n* = 100; public *n* = 47, 13%; private *n* = 53, 14%; *p* = 0.64) reported correctly that when people take too many antibiotics, it is not their body that becomes resistant to them. Persons with higher education levels had better knowledge scores (matric or below = 50%, s.d. 18%; tertiary education = 62%, s.d. 20%; *p* ≤ 0.001).

Private practice patients scored higher on 12 of the 15 knowledge questions. More public practice patients (*n* = 326, 86%) knew that antibiotics were used to treat bacterial infections in comparison to private sector patients (*n* = 300, 77%); however, only a small percentage believed that antibiotics are not good for treating viruses (all *n* = 190, 24%; public *n* =36, 10%; private *n* = 154, 39%; *p* ≤ 0.001). Public sector patients scored lowest on the question around antibiotics being used to treat viruses. Private practice patients scored lowest on the question about the human body becoming resistant to antibiotics and both groups scored low on the question of whether or not flu was caused by bacteria (Table 2).

**TABLE 2:** Knowledge questions, correct answers by practice type (*p*-values from chi-square tests).

Variable	All (*N* = 782)		Public (*N* = 379)		Private (*N* = 403)		Chi-square test
	*n*	%	*n*	%	*n*	%	
Antibiotics are not good for treating germs called viruses	190	24	36	10	154	39	< 0.001
Antibiotics are good for treating germs called bacteria	626	80	326	86	300	77	0.001
Colds, flu and runny nose are not usually caused by germs called bacteria	142	18	33	9	109	28	< 0.001
Our body has ‘good’ bacteria which keeps us healthy	551	70	229	61	322	82	< 0.001
Antibiotics can be harmful by killing the ‘good’ bacteria	412	53	169	45	243	62	< 0.001
Antibiotics do not only kill the ‘bad’ bacteria that make you sick	264	34	51	13	213	54	< 0.001
People can be allergic to antibiotics	638	82	297	79	341	86	0.006
Antibiotics can cause diarrhoea	428	55	140	37	288	74	< 0.001
All people with a sore throat do not need antibiotics	398	51	108	29	290	75	< 0.001
Antibiotics are needed for most sexually transmitted infections	361	46	203	54	158	40	< 0.001
People with pneumonia need antibiotics	509	65	242	64	267	68	0.19
Antibiotics are needed for most urine infections	498	64	270	72	228	58	< 0.001
Antibiotics do have side-effects	514	66	210	53	313	79	< 0.001
When people take too many antibiotics their body does not become resistant to them	100	13	47	13	53	14	0.64
When people take too many antibiotics the germs becomes resistant to them	481	62	213	56	268	69	< 0.001

### Knowledge scores, practice type and associations with antibiotic resistance attitudes

Table 3 explores the associations between knowledge categories, practice types and beliefs and behaviours. Public practice patients reported feeling relieved when being prescribed antibiotics because they felt this meant that the prescriber realised they were sick (low knowledge scorers (low) *n* = 243, 84%; high knowledge scorers (high) *n* = 70, 81%) and feeling happy as they felt the visit was justified (low *n* = 222, 77%; high *n* = 71, 82%). They noted that they believed it is most important to have antibiotics when one is very sick (low *n* = 195, 67%; high *n* = 71, 82%). Both public sector knowledge score groups exhibited some protective behaviours such as finishing the course of antibiotics (low *n* = 269, 92%; high *n* = 84, 97%). Public sector high knowledge scorers more frequently noted that taking antibiotics is not about the severity of sickness, but the kind of disease (low *n* = 252, 87%; high *n* = 83, 95%). The main difference between knowledge scoring groups in public sector patients was in the protective factors such as the belief that antibiotics can upset the body’s natural balance (low 41%; high 78%; *p* ≤ 0.01) and that ABR will be costly to the world (low 56%; high 80%; *p* ≤ 0.001).

**TABLE 3:** Beliefs and behaviours between low and high knowledge scorers in both the public and private sector

Harmful beliefs	Total *N* = 782		Public (*N* = 379)					Private (*N* = 403)				
			Low knowledge (*N* = 291) agree/true		High knowledge (*N* = 87) agree/ true		Chi-square test (high vs. low)	Low knowledge (*N* = 154) agree/true		High knowledge (*N* = 243) agree/true		Chi-square test (high vs. low)
	*n*	%	*n*	%	*n*	%		*n*	%	*n*	%	
If people demand an antibiotic, the doctor or nurse should give it to them	193	25	108	37	38	44	0.02	23	15	24	10	< 0.001
Antibiotics should be available without a prescription	137	18	66	23	24	28	0.02	22	15	25	10	< 0.001
Scientists will discover new antibiotics if the current ones stop working	425	54	191	66	70	80	0.01	57	39	107	45	0.32
I have been to the clinic/ doctor specifically because I wanted an antibiotic	301	38	134	46	46	53	0.28	39	27	82	34	0.17
I have taken antibiotics that were prescribed for a friend or family member	122	16	62	21	23	26	0.33	14	11	23	10	0.90
I have saved unused antibiotics to use at a later time	161	21	70	24	27	31	0.20	22	16	42	18	0.63
I have given my antibiotics to a friend or family member	130	17	59	20	25	29	0.10	21	16	25	11	0.20
I have exaggerated my symptoms to get antibiotics	102	13	50	17	22	25	0.10	16	12	14	6	0.05
When I am prescribed antibiotics I feel relieved that the doctor or nurse realises I am sick	463	29	243	84	70	81	0.43	62	48	88	40	0.13
When I am prescribed antibiotics I feel happy because my visit was justified	407	52	222	77	71	82	0.35	45	36	69	33	0.56
It is most important to have antibiotics when I have been sick for a long time	474	61	195	67	69	79	0.03	83	58	127	53	0.35
It is most important to have antibiotics when I am very sick	487	62	195	67	71	82	0.01	88	62	133	56	0.23
It is most important to have antibiotics when I tried simple remedies but they did not work	427	55	171	59	63	72	0.021	69	49	124	52	0.50
**Protective beliefs**												
Antibiotics can upset your body’s natural balance	491	62	119	41	67	78	< 0.001	81	53	224	93	< 0.001
It is important for me to finish the course of antibiotics I have been prescribed	707	90	269	92	84	97	0.39	117	77	237	98	< 0.001
Antibiotics are strong and you should only take them when you really need them	637	81	238	82	79	91	0.1	99	66	221	91	< 0.001
Antibiotics will work less well in future if we overuse them now	473	61	161	55	63	72	0.01	69	47	180	75	< 0.001
Antibiotic resistance is likely to be very costly to the world	456	58	163	56	70	80	< 0.001	52	37	171	72	< 0.001
When I am prescribed antibiotics I feel annoyed because too many antibiotics are being prescribed	174	22	60	21	29	34	0.013	32	26	53	26	0.97
When I am prescribed antibiotics I feel worried because I prefer not to take antibiotics unless absolutely necessary	415	53	162	56	57	66	0.091	69	54	127	60	0.34
It is most important to have antibiotics when the doctor or nurse thinks I have a bacterial infection	618	79	259	89	80	92	0.43	92	64	187	78	0.002
Taking antibiotics is not about how sick I am, it depends what kind of illness I have	654	84	252	87	83	95	0.023	105	74	214	89	< 0.001

Private practice patients believed that antibiotics should be used when very sick (low *n* = 88, 92%; high *n* = 133, 56%) and when sick for a long time (low *n* = 83, 58%; high *n* = 133, 56%). Both low and high knowledge scoring groups thought it was important to finish a course of antibiotics (low *n* = 117, 77%; high *n* = 237, 98%), those with below mean scores believed it was not about the severity but the kind of illness (*n* = 105, 74%) and those with high knowledge scores believed antibiotics can upset the body’s natural balance (*n* = 224, 93%). The two main differences in beliefs of private patients are the belief that antibiotics need to be used conservatively (low 47%; high 75%; *p* ≤ 0.001) and that ABR will be costly for the world (low 37%; high 72%; *p* ≤ 0.001).

Public practice patients with high knowledge scores were more likely to report negative and protective behaviours; those with low knowledge scores were less likely to exhibit negative beliefs and behaviours. This pattern is not seen in private practice patients. The high knowledge scorers in this group were marginally less likely to report negative beliefs and behaviours and more likely to report protective behaviours.

### Alternative to prescribing

Thirty-eight per cent (*n* = 295) of patients reported ever being told that antibiotics were not needed for themselves or their child (public *n* = 100, 27%; private *n* = 195, 53%; *p* ≤ 0.001). Alternative strategies are needed in order to reduce the amount of unnecessary prescriptions of antibiotics. One hundred and forty-four patients (18%) would only have been satisfied if antibiotics were prescribed after a visit to the doctor (public *n* = 92, 24%; private *n* = 52, 13%; *p* ≤ 0.001), but the respondents also noted several acceptable alternatives. Three hundred and fifty-nine (46%) patients reported that they would be happy with advice on what to buy over the counter (public *n* = 121, 32%; private *n* = 238, 59%; *p* ≤ 0.001) and 271 would be satisfied with information or reassurance about the illness (all = 35%; public *n =* 94, 5%; private *n* = 177, 44%; *p* < 0.001) (Table 4).

**TABLE 4:** Acceptable alternatives to prescribing.

Variable	Total (*N* = 782)		Public (*N* = 379)		Private (*N* = 403)		Chi-square test
	*n*	%	*n*	%	*n*	%	
Advice on what to buy over the counter	359	46	121	32	238	59	< 0.001
A different medicine that is only available on prescription	235	30	101	27	134	33	0.044
A homeopathic remedy	86	11	63	17	23	6	< 0.001
An injection of vitamins	236	30	92	24	144	36	< 0.001
Referral to a traditional healer	51	7	28	7	23	6	0.34
Information and reassurance about the illness	271	35	94	25	177	44	< 0.001
I would only be satisfied if I was given an antibiotic	144	18	92	24	52	13	< 0.001

Thirty-nine per cent (*n* = 301) of all respondents had at some point visited the doctor specifically so antibiotics could be prescribed (public *n* =180, 48%; private *n* =121, 32%; *p* ≤ 0.001). Of these, 259 (86%) said it was because they thought the antibiotics would make them better, 218 (73%) said they thought antibiotics worked well because you only get them from a nurse or a doctor, and 202 (67%) said they believed the antibiotics would make them be able to get back to work sooner. The main reasons for wanting to take antibiotics included having a bacterial infection (total *n* = 618, 79%; public *n* = 339, 90%; private *n* = 279, 73%; *p* ≤ 0.001), being sick for a long time (total *n* = 474, 61%; public *n* = 264, 70%; private *n* = 210, 55%; *p* ≤ 0.001) and being very sick (total = 487, 62%; public *n* = 266, 71%; private *n* = 221, 58%; *p* ≤ 0.001).

## Discussion

Our study showed that the majority of patients are aware of the threat of ABR and that antibiotics will work less well in future if over prescribed now. However, it is apparent that patients in both the public and private sector continue to have many misconceptions about antibiotics. Public practice patients were older and had lower levels of education; this is indicative of the inequality of the health system in South Africa.^33,34,35^ The mean knowledge scores around ABR were suboptimal in both groups, and we identified that poor knowledge was associated with harmful perceptions and behaviours. A high proportion of patients reported they would be satisfied with alternatives to be prescribed.

Beliefs about ABR are different between low and high scoring knowledge groups in both practice types. High knowledge scorers from public practices are more likely to exhibit negative behaviours but also protective behaviours. Low knowledge scorers from private practices exhibit negative behaviours and high knowledge scorers exhibit protective behaviours. These are novel findings, which can create the foundation for activities around patient education programmes for ABR stewardship throughout South Africa.

Our results were similar to the McCoullough et al.’s systematic review of 54 studies on ABR which showed that 88% of the study population believed ABR referred to changes in the human body.^14^ In our study, this proportion was 72%. Our study showed that 61% of respondents believed that ABR was a problem, and 79% of patient respondents reported this in a similar study in Britain.^36^ Public practice patients were slightly more concerned about the cost of AMR in comparison to private patients. Thirty-six per cent of participants in both studies did not know that antibiotics do not work for common colds and sore throats,^36^ a finding corroborated by other studies.^4,16,37,38,39,40,41^ Just over half of the respondents reported being worried when prescribed antibiotics which is contradictory to other findings in our study such as the noted destructive behaviours and the lack of understanding of the differences between viral and bacterial infections. Private practice patients were more likely to be told antibiotics were not needed for them (53% vs. 27%), which could show that private practice patients are more likely to request antibiotics at appointments and are thus more likely denied this form of treatment. This pressure from patients has been widely reported and is noted as one of the leading causes of the overuse of antibiotics.^4,14,20,42,43,44,45,46^ This could be a focus area for interventions.

Our study shows that patients attending public health facilities, have a sense of relief when given an antibiotic as the prescription is a validation of their illness. This is problematic as this need for validation could override the benefits of knowledge about ABR showing that knowledge does not necessarily translate into positive behavioural changes. Our study did show acceptable alternatives to prescribing, such as giving advice on what to buy over the counter, and these methods, along with providing patients with accurate information about ABR, would need to be utilised in order to make sure patients feel their visits have been validated.

Our study shows that high knowledge scores are not exclusively related to positive behaviours and beliefs. This is similar to other studies which showed that those with higher knowledge levels were no less likely to be prescribed an antibiotic.^37^ Our results are similar to findings from previous studies showing that any planned interventions may be partially effective if focusing on increasing awareness in patient populations, but this cannot be the sole intervention.

## Limitations

We must highlight that this survey relied on convenience sampling, and thus, the results might not be generalisable to the general population. All participants were from urban settings, and so the KAP of rural patients was not explored. Selection bias could have occurred as only literate participants would have opted in to complete the survey. People with certain traits may have been more prone to accepting, such as those who felt confident about their knowledge of ABR. A further selection bias factor is that only patients who were already at health care practices were requested to complete the survey. These persons would possibly be different to the general public in that they sought health care and therefore might have more knowledge of available treatments such as antibiotics. There was also a possibility that those coming to the clinic to collect the medication were not the patients themselves, but the caretakers or family members of the patients; this could influence the answers given. There was no mechanism in place to ensure that people did not complete the survey more than once, but given that the study took some time to complete, and there was no specific incentive to participate, double completions were unlikely. Social desirability bias could be introduced as the accuracy of answers could not be assessed, because they are self-reported, but this was minimised as the surveys were anonymous.

## Potential intervention points

A range of intervention points could be utilised including education programmes for patients, shortened and delayed prescriptions and alternatives to be prescribed. Education has been successful in a range of settings,^42^ and our study indicated that high knowledge scorers generally exhibit more protective beliefs and behaviours than those with low knowledge scores, which could show that education programmes could be a useful intervention in South Africa to encourage positive behavioural changes. Similar studies have suggested educating patients about the consequences of the misuse of antibiotics and what diseases actually require antibiotics,^11^ and on not reusing and recycling antibiotics and not to self-medicate,^37^ issues seen in our findings. Educational programmes should be run with caution as some studies have reported that such interventions led to more frequent requests for antibiotics.^45^ In the South African setting, these educational programmes could take the form of patient and prescriber communication tools, radio adverts and online media, which is becoming a widely used resource in South Africa.

As knowledge is not always associated with positive behaviours, different intervention options should be sought such as delaying antibiotic prescriptions.^37^ This has been shown to be effective in decreasing antibiotic use;^47^ however, this method has led to some patient dissatisfaction.^48^ A further reportedly effective intervention would be to shorten the course of antibiotics prescribed,^37^ which would reduce the risk of patients having leftover antibiotics and thus prevent harmful behaviours such as sharing antibiotics with friends and family and saving antibiotics for future use which was reported in a systematic review^11^ and in our study.

This study has yielded information which may assist in using alternatives to be prescribed such as different treatments (vitamins, over the counter medications). Our study has shown that these alternatives would be acceptable in the South African setting. These alternatives should be shared with practitioners, and future research should focus on the most effective alternatives, which should then become common practice.

## Conclusion

Our study demonstrates that there are differences in the attitudes, perceptions and behaviours of public and private health care patients of antibiotic use and resistance. Our findings also illustrate that those with greater knowledge of ABR tend to exhibit more protective behaviours and beliefs. The study has shown a range of intervention points that could be effective in South Africa including education programmes, shortened and delayed prescriptions, and alternatives to be prescribed. We encourage further in-depth qualitative research with patient groups to better understand the required messages for ABR interventions and the effectiveness of existing patient education strategies around the topic.

## Appendix 1: Questionnaire

### Knowledge attitudes and perceptions of antibiotic use among patients in primary care in South Africa

Gender: Male/ Female

Age: Under 25/ 26-35/ 36-45/ 46-55/56-65/ Above 65

Type of clinic: Private GP/ government clinic

Highest level of education: Grade 1 – 8/ Grade 9-11/ Matric/ University degree/ Postgraduate degree

### Knowledge

1. **Which of the following is true? (True/False/Unsure)**
  - Antibiotics are good for treating germs called viruses.
  - Antibiotics are good for treating germs called bacteria.
  - Colds, flu and runny nose are usually caused by germs called bacteria.
  - Our body has ‘good’ bacteria which keeps us healthy.
  - Antibiotics can be harmful by killing the ‘good’ bacteria.
  - Antibiotics only kill the ‘bad’ bacteria that make you sick.
  - People can be allergic to antibiotics.
  - Antibiotics can cause diarrhoea.
  - All people with a sore throat need antibiotics.
  - Antibiotics are needed for most sexually transmitted infections (STI’s).
  - People with pneumonia need antibiotics.
  - Antibiotics are needed for most urine infections.
2. **Which of the following are true? (True/False/Unsure)**
  - Antibiotics do not have side effects.
  - Antibiotics can upset your body’s natural balance.
  - It’s important for me to finish the course of antibiotics I have been prescribed.
  - If people demand an antibiotic, the doctor/nurse should give it to them.
  - Antibiotics should be available without a prescription.
  - Antibiotics are strong and you should only take them when you really need them.
3. **With regard to antibiotic resistance, which of the following are true (True/False/Unsure)**
  - When people take too many antibiotics their body becomes resistant to them.
  - When people take too many antibiotics the germs becomes resistant to them.
  - Antibiotics will work less well in future if we overuse them now.
  - Antibiotic resistance is likely to be very costly to the world.
  - Scientists will discover new antibiotics if the current ones stop working.
4. **When is it most important to have antibiotics? (True/False/Unsure)**
  - When the doctor or nurse thinks I have a bacterial infection.
  - When I have been sick for a long time.
  - When I am very sick.
  - When I tried simple remedies but they did not work.
  - It is not about how sick I am, it depends what kind of illness I have.
5. **Have you ever been to the clinic specifically because you wanted an antibiotic for you or your child?**
  - Yes - Continue to question 6
  - No - go to question 7
6. **If yes, what were your beliefs about antibiotics? (Tick all that apply)**
  - I thought antibiotics would make me or my child better.
  - I think antibiotics work well because you can only get them from the doctor or nurse.
  - Antibiotics are harmless so it’s better to be safe.
  - I believed they would help me get back to work sooner.
  - It is good to have a stock of antibiotics at home for when you need them.
  - I am a woman of child bearing age and am confident I can self-diagnose cystitis.
7. **Have you ever been told that antibiotics are not needed for you or your child? If yes, what did the doctor or nurse do? (tick all that apply)**
  - Explained that I or my child had a virus and antibiotics will not help.
  - Explained that antibiotics have side effects which could make me feel worse.
  - Explained what to expect in terms of symptom resolution.
  - I was given written information about why antibiotics would not help and may cause harm.
  - I was given written information about what to expect in terms of symptom resolution.
  - I was told I or my child needed follow-up if symptoms did not improve.
  - I was prescribed symptomatic relief.
  - I was given a delayed prescription for antibiotics (a prescription I could only collect after a few days if I wasn’t better).
8. **If you or your child had a cough or cold and the doctor or nurse said you did not need antibiotics, which of the following is likely to leave you satisfied with the consultation? (tick all that apply)**
  - Advice on what to buy over the counter.
  - A different medicine that is only available on prescription.
  - A homeopathic remedy.
  - An injection of vitamins.
  - Referral to a traditional healer.
  - Information and reassurance about the illness.
  - I would only be satisfied if I was given an antibiotic.
9. **Which of the following apply to you? (True/False/Unsure)**
  - I have taken antibiotics that were prescribed for a friend or family member.
  - I have saved unused antibiotics to use at a later time.
  - I have given my antibiotics to a friend or family member.
  - I have exaggerated my symptoms to get antibiotics.
10. **How do you feel when the doctor or nurse prescribes antibiotics (Yes/Unsure/No)**
  - Relieved that the doctor or nurse realises I am sick.
  - Happy because my visit was justified.
  - Annoyed because too many antibiotics are being prescribed.
  - Worried because I prefer not to take antibiotics unless absolutely necessary.
